# EVIDENCE-BASED REVIEWS & RECOMMENDATIONS FROM THE BRAZILIAN SOCIETY OF SHOULDER AND ELBOW SURGERY: HYALURONIC ACID INFILTRATION FOR ELBOW EPICONDYLITIS IN ADULTS

**DOI:** 10.1590/1413-785220263401e303257

**Published:** 2026-08-31

**Authors:** Gustavo de Mello Ribeiro Pinto, Leonardo Zanesco, Marcus Vinícius Galvão Amaral, Fábio Teruo Matsunaga, João Artur Bonadiman, João Felipe de Medeiros, Marcelo Costa de Oliveira Campos, Eduardo Angeli Malavolta

**Affiliations:** 1 Universidade do Estado do Rio de Janeiro (UERJ), Rio de Janeiro, RJ, Brazil.; 2 Universidade de Sao Paulo, Faculdade de Medicina, Hospital das Clinicas HCFMUSP, Sao Paulo, SP, Brazil.; 3 Hcor, Sao Paulo, Brazil.; 4 Centro de Cirurgia do Ombro e Cotovelo, Instituto Nacional de Traumatologia e Ortopedia Jamil Haddad, Rio de Janeiro, RJ, Brazil.; 5 Universidade Federal de Sao Paulo, Escola Paulista de Medicina, Departamento de Ortopedia, Grupo de Ombro e Cotovelo, Sao Paulo, SP, Brazil.; 6 Instituto de Ortopedia e Traumatologia, Hospital Sao Vicente de Paulo, Passo Fundo, RS, Brazil.; 7 Universidade Federal do Rio Grande do Norte, Natal, RN, Brazil.

**Keywords:** Lateral Epicondylitis, Tennis Elbow, Hyaluronic Acid, Corticosteroid, Injections, Elbow, Epicondilite Lateral, Cotovelo de Tenista, Ácido Hialurônico, Corticosteroide, Injeções, Cotovelo

## Abstract

**Introduction:**

Elbow epicondylitis is a common tendinopathy in adults, and infiltration is frequently used when initial conservative treatment fails. Hyaluronic acid and corticosteroids are among the most widely used injectable agents, but their comparative effectiveness remains uncertain.

**Methods:**

We performed an evidence-based review of randomized controlled trials and systematic reviews comparing hyaluronic acid with corticosteroid injections for adult elbow epicondylitis. PubMed, Embase, the Cochrane Library, and BVS were searched through August 2025. Main outcomes were pain and elbow function; risk of bias and certainty of evidence were assessed using Cochrane and GRADE approaches.

**Results:**

Of 94 records, three studies met the eligibility criteria (one systematic review and two randomized trials), all focused on lateral epicondylitis. Very low-quality evidence suggests that hyaluronic acid provides greater improvement in pain and function than corticosteroids from 8 weeks to 6 months in acute cases, whereas in chronic lateral epicondylitis, corticosteroid injections appear more effective than hyaluronic acid for pain relief and functional recovery up to 6 weeks.

**Conclusions:**

Current evidence, though very limited and at high risk of bias, supports preferential use of corticosteroids for short-term symptom control and hyaluronic acid for medium- to long-term pain and functional improvement in adult lateral epicondylitis. ***Level of evidence II; Systematic review.***

## RESUMO

**Introdução:**

A epicondilite do cotovelo é uma tendinopatia frequente em adultos, e infiltrações são frequentemente utilizadas quando o tratamento conservador inicial falha. Ácido hialurônico e corticosteroides estão entre os agentes injetáveis mais utilizados, porém sua efetividade comparativa permanece incerta.

**Métodos:**

Foi realizada uma revisão baseada em evidências de ensaios clínicos randomizados e revisões sistemáticas que compararam infiltrações de ácido hialurônico com corticosteroides em adultos com epicondilite do cotovelo. As bases de dados PubMed, Embase, Cochrane Library e BVS foram pesquisadas até agosto de 2025. Os principais desfechos foram a dor e a função do cotovelo; o risco de viés e a certeza da evidência foram avaliados segundo a metodologia Cochrane e a abordagem GRADE.

**Resultados:**

Dos 94 registros, três estudos preencheram os critérios de elegibilidade (uma revisão sistemática e dois ensaios randomizados), todos focados em epicondilite lateral. Evidência de qualidade muito baixa sugere que o ácido hialurônico proporciona maior melhora da dor e da função do que os corticosteroides entre 8 semanas e 6 meses em casos agudos, enquanto, na epicondilite lateral crônica, as infiltrações com corticosteroides parecem mais eficazes do que o ácido hialurônico para alívio da dor e recuperação funcional até 6 semanas.

**Conclusão:**

A evidência disponível, embora muito limitada e com alto risco de viés, apoia o uso preferencial de corticosteroides para controle sintomático em curto prazo e do ácido hialurônico para melhora da dor e da função em médio e longo prazos na epicondilite lateral em adultos. ***Nível de Evidência II; Revisão sistemática.***

## RECOMMENDATION

There is evidence, although of very low quality, that hyaluronic acid infiltration is more effective than corticosteroid infiltration for the treatment of acute lateral epicondylitis of the elbow in adults regarding pain and functional outcomes between 8 weeks and 6 months after infiltration (Level of Evidence I–II).

There is also evidence, although of very low quality, that corticosteroid infiltration is more effective than hyaluronic acid infiltration for the treatment of chronic lateral epicondylitis of the elbow in adults, regarding pain and function up to 6 weeks after infiltration (Level of Evidence I–II). However, studies with greater methodological precision are still needed to clarify this issue.

The SBCOC Consensus Project recommends that, when conventional conservative treatment for epicondylitis fails, hyaluronic acid infiltration may be performed to improve pain and function in the medium and long term, while corticosteroids should be used to improve these outcomes in the short term, in cases of acute pain. Additionally, attention should be given to local and systemic complications associated with corticosteroid use, which represent a disadvantage compared to hyaluronic acid.

## INTRODUCTION

Elbow epicondylitis is a degenerative inflammatory disorder caused by repetitive overload (microtrauma) of the tendons originating from the medial and lateral epicondyles of the humerus, leading to collagen degeneration, neovascularization, and pain. It is divided into lateral epicondylitis (tennis elbow), which mainly affects the extensor carpi radialis brevis tendon and is the most common type; and medial epicondylitis (golfer's elbow), which affects the origin of the wrist flexor tendons. The estimated annual incidence is 5 to 7 cases per 1000 people.^1–3^

Conservative treatment is effective in most cases. Among therapeutic options are physical therapy, analgesic and anti-inflammatory medications, activity modification, immobilization, acupuncture, stretching, shock wave therapy, and infiltrations.^3–6^

By all of the available infiltration options, corticosteroids and hyaluronic acid are the most commonly used. Corticosteroid infiltration has a theoretical anti-inflammatory and analgesic effect, improving pain and function in the short term (up to 8 weeks); however, these effects are not maintained in the medium and long term (beyond 3–6 months). The main complications of corticosteroid use are post-infiltration pain, skin atrophy, tendon degeneration, risk of tendon rupture, and systemic complications.^6–9^

Hyaluronic acid infiltration, in theory, improves local viscoelasticity, and also has analgesic and tissue repair stimulation effects. Literature reports demonstrate improvement in pain and function in the short and medium term (up to 12 months), even showing superiority over corticosteroids in some studies. Potential complications include infection, foreign body reaction, and allergy. On the other hand, it reduces the need for repeated infiltrations and has fewer local adverse effects compared to corticosteroids, and no known systemic reactions.^7,9–11^

For these reasons, hyaluronic acid infiltration is increasingly used by physicians in the treatment of epicondylitis. The present consensus aims to review current literature to assess whether hyaluronic acid infiltration is more effective than corticosteroid infiltration in terms of pain and functional outcomes.^10–15^

This evidence review was developed within a collaborative initiative between Cochrane and the Brazilian Society of Shoulder and Elbow Surgery (SBCOC). The aim of this partnership is to synthesize the best available evidence using rigorous, transparent methods and to translate it into clear, practical recommendations for clinical practice.

### Research Question

Is hyaluronic acid infiltration more effective than corticosteroid infiltration for the treatment of elbow epicondylitis in adults?

## METHODS

- Databases searched: PubMed, Embase, Cochrane Library, and BVS.
- Descriptors: As described in Appendix 1.
- Search period: Until August 2025.
- Included studies: according to the highest level of evidence, prioritizing systematic reviews (SRs) with high methodological quality and recency. In the absence of SRs, randomized controlled trials (RCTs) were included, followed by observational studies and, lastly, case series.

## RESULTS

**Figure 1:**
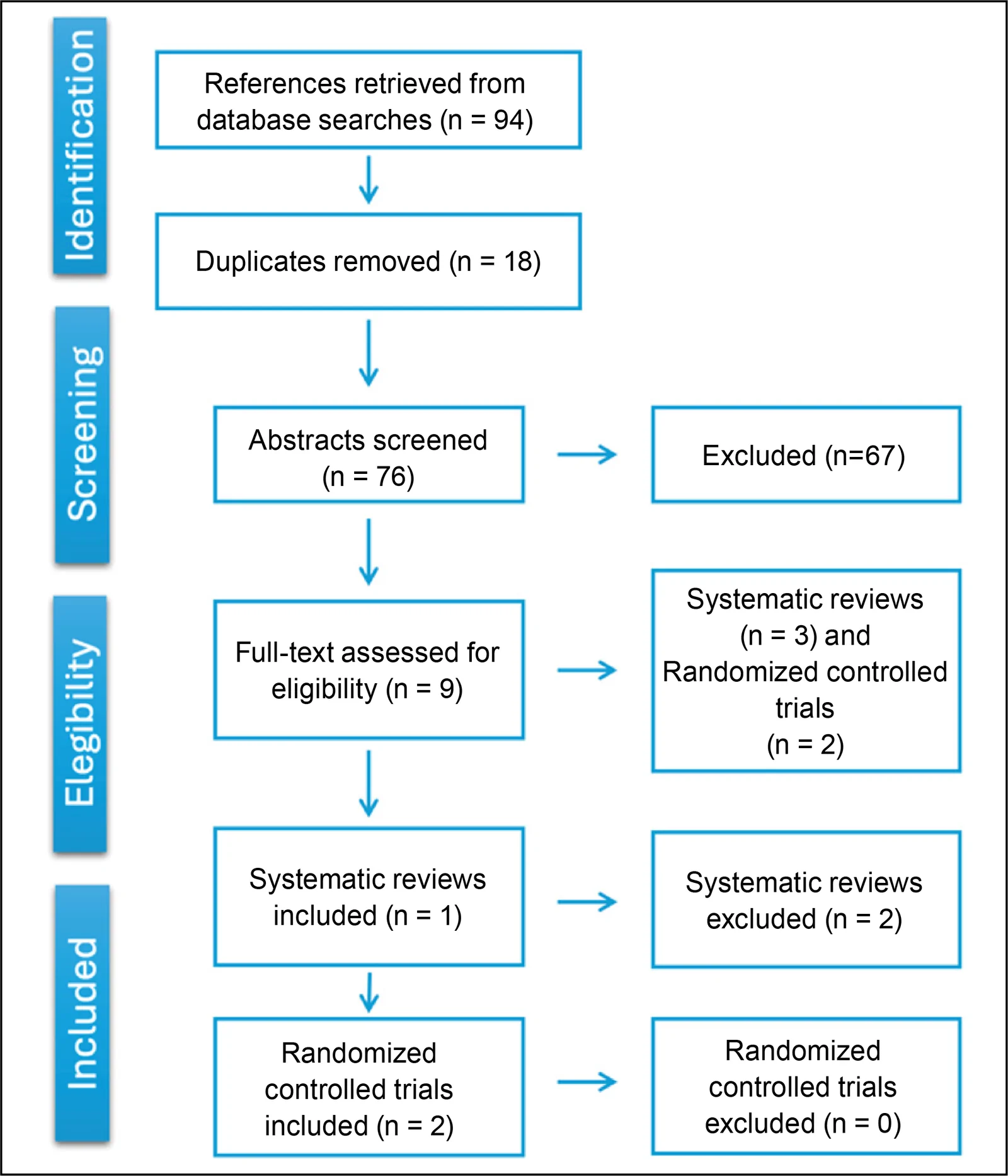
Flow diagram of the literature search.

The search found 94 references (33 PubMed, 23 Embase, 34 Cochrane Library, 4 BVS). Nine full-text articles were selected for evaluation, of which three were excluded for not including both comparisons.^18–20^ Three others^21–23^ were excluded for not being RCTs or SRs, leaving three studies included in this review.^6,14,15^ (Figure 1).

### Included studies

In a systematic review, Krogh et al. (2012) analyzed the effectiveness of infiltrations in lateral elbow epicondylitis regarding pain outcomes and adverse effects. A total of 1381 participants (17 RCTs) were included, evaluating injections of corticosteroids, botulinum toxin, platelet-rich plasma, autologous blood, hyaluronic acid, prolotherapy, polidocanol, glycosaminoglycan polysulfate, and placebo. In an indirect meta-analysis, corticosteroids (8 trials; 464 participants) were not more effective than placebo in pain reduction after 8 weeks (SMD −0.04; 95% CI −0.45 to 0.35). Hyaluronic acid was assessed in one study (331 participants) and was considered more effective than placebo (SMD −5.58; 95% CI −6.35 to −4.82) after 8 weeks for pain. The authors noted that most studies had high risk of bias thereby the results should be interpreted cautiously, and also the outcomes were not evaluated before 8 weeks.

Tosun et al. (2015) conducted an RCT (57 participants) comparing a single infiltration with hyaluronic acid with chondroitin sulfate (25 participants) versus a single corticosteroid infiltration (32 participants). The study concluded that hyaluronic acid with chondroitin sulfate infiltration was more effective (clinically and statistically) regarding functional outcomes at 3 and 6 months after the injection, and for pain at 6 months. The main limitations were small sample size, lack of control group, short follow-up, and absence of cost-effectiveness analysis^14^.

Yalcin et al. (2022) conducted an RCT with 80 participants with chronic lateral epicondylitis, in which 40 participants received hyaluronic acid infiltration and 40 received triamcinolone infiltration—assessed at 6 and 12 weeks after the procedure. Outcomes included pain and function according to the Q-DASH questionnaire, visual analog scale (VAS), and handgrip strength measured by dynamometry. The authors concluded that hyaluronic acid did not demonstrate superiority over the corticosteroids, and corticosteroid infiltration was more effective for pain control and function recovery at 6 weeks in chronic cases^15^.

The general characteristics of the evaluated studies and the quality of evidence according to the GRADE system^16,17^ are presented in Tables 1 and 2, respectively.

**Table 1:** Characteristics of the included studies.

Included papers	Methods	Included papers characteristics	Sample size	Protocol previous published
Krogh et al. (2012)	Systematic Review	17 (RCT) 8 with corticosteroid 1 with hyaluronic acid	1381	No
Tosun et al. (2015)	Randomized clinical trial	--	57	No
Yalcin et al.(2022)	Randomized clinical trial	- -	80	No

**Table 2:** Quality of evidence of the included studies (GRADE).

Outcome	Number of studies	Quality of evidence
Pain	3 studies	Very low^1^,^2^,^3^,^4^
Elbow function (different scores)	2 studies	Very low^1^,^2^,^3^,^4^

1 Limited sample size

2 Imprecision (Large IC)

3 Risk of bias of the included study

4 No previous protocol. Note: GRADE (Grading of Recommendations Assessment, Development and Evaluation) is a tool used to evaluate and measure the quality of evidence. It is based in 5 pillars: risk of bias, imprecision, inconsistency, direct evaluation of the outcome and publication bias. Being so the quality if evidence is classified in very low, low, moderate and high quality ^16,17^.

## CONCLUSION

There is very low-quality evidence showing that hyaluronic acid infiltration is more effective than corticosteroid infiltration for treating acute elbow epicondylitis in adults, regarding pain and function up to 6 months. There is very low-quality evidence showing that corticosteroid infiltration is more effective than hyaluronic acid infiltration for treating elbow epicondylitis in adults, regarding pain and function up to 6 weeks. In summary, corticosteroid use appears more effective for early outcomes (up to 6 weeks), while hyaluronic acid demonstrates greater effectiveness for pain and function after 8 weeks.

## Data Availability

The data underlying the research text are contained in the manuscript.

## Appendix 1 Database search strategies.

**Table:**

**PUBMED**
#1 "Tennis Elbow"[Mesh] OR (Elbow, Tennis) OR (Tennis Elbows) OR (Lateral Epicondylitis) OR (Epicondylitides, Lateral) OR (Epicondylitis, Lateral) OR (Lateral Epicondylitides) OR (Epicondylitis, Lateral Humeral) OR (Epicondylitides, Lateral Humeral) OR (Humeral Epicondylitides, Lateral) OR (Humeral Epicondylitis, Lateral) OR (Lateral Humeral Epicondylitides) OR (Lateral Humeral Epicondylitis) OR "Elbow Tendinopathy"[Mesh] OR (Elbow Tendinopathies) OR (Tendinopathies, Elbow) OR (Tendinopathy, Elbow) OR (Medial Epicondylitis) OR (Epicondylitis, Medial) OR (Medial Epicondylitides) OR (Golfer's Elbow) OR (Golfers Elbow)
#2 "Injections, Intra-Articular"[Mesh] OR (Injection, Intra-Articular) OR (Intra-Articular Injection) OR (Intra Articular Injection) OR (Articular Injection, Intra) OR (Articular Injections, Intra) OR (Injection, Intra Articular) OR (Injections, Intra Articular) OR (Intra Articular Injections) OR (Intraarticular Injection) OR (Injection, Intraarticular)
#3 "Hyaluronic Acid"[Mesh] OR (Acid, Hyaluronic) OR (Amvisc) OR (Luronit) OR (Hyvisc) OR (Hyaluronan) OR (Sodium Hyaluronate) OR (Hyaluronate, Sodium) OR (Hyaluronate Sodium) OR (Healon) OR (Etamucine) OR (Amo Vitrax) OR (Vitrax, Amo) OR (Biolon)
#4 "Adrenal Cortex Hormones"[Mesh] OR (Hormones, Adrenal Cortex) OR (Corticoid) OR (Adrenal Cortex Hormone) OR (Cortex Hormone, Adrenal) OR (Hormone, Adrenal Cortex) OR (Corticosteroid) OR (Corticoids) OR (Corticosteroids)
#5 #3 OR #4
#6 #1 AND #2 AND #5
Filters applied: Randomized Controlled Trial, Systematic Review = 33

**Table:**

**COCHRANE**
#1 (Tennis Elbow) OR (Elbow, Tennis) OR (Tennis Elbows) OR (Lateral Epicondylitis) OR (Epicondylitides, Lateral) OR (Epicondylitis, Lateral) OR (Lateral Epicondylitides) OR (Epicondylitis, Lateral Humeral) OR (Epicondylitides, Lateral Humeral) OR (Humeral Epicondylitides, Lateral) OR (Humeral Epicondylitis, Lateral) OR (Lateral Humeral Epicondylitides) OR (Lateral Humeral Epicondylitis) OR (Elbow Tendinopathy) OR (Elbow Tendinopathies) OR (Tendinopathies, Elbow) OR (Tendinopathy, Elbow) OR (Medial Epicondylitis) OR (Epicondylitis, Medial) OR (Medial Epicondylitides) OR (Golfer's Elbow) OR (Golfers Elbow).
#2 (Injections, Intra-Articular) OR (Injeção Intra-Articular) OR (Injeção Intra-Articular) OR (Injeção Intra-Articular) OR (Injeção Articular, Intra) OR (Injeções Articulares, Intra) OR (Injeção Intra-Articular) OR (Injeções Intra-Articulares) OR (Injeções Intra-Articulares) OR (Injeção intra-articular) OR (Injeção Intra-articular) OR (Injeções Intra-Articulares) OR (Injeções Intra-articulares) OR (Injeções intra-articulares).
#3 (Hyaluronic Acid) OR (Acid, Hyaluronic) OR (Amvisc) OR (Luronit) OR (Hyvisc) OR (Hyaluronan) OR (Sodium Hyaluronate) OR (Hyaluronate, Sodium) OR (Hyaluronate Sodium) OR (Healon) OR (Etamucine) OR (Amo Vitrax) OR (Vitrax, Amo) OR (Biolon).
#4 (Adrenal Cortex Hormones) OR (Hormones, Adrenal Cortex) OR (Corticoid) OR (Adrenal Cortex Hormone) OR (Cortex Hormone, Adrenal) OR (Hormone, Adrenal Cortex) OR (Corticosteroid) OR (Corticoids) OR (Corticosteroids).
#5 #3 OR #4.
#6 #1 AND #2 AND #5 = 34

**Table:**

**EMBASE**
#1 'tennis elbow'/exp OR 'bursitis, radiohumeral' OR 'elbow, tennis' OR 'epicondylatgia humeri' OR 'epicondylitis humeri' OR 'epicondylitis lateralis' OR 'epicondylitis, external humeral' OR 'epicondylitis, radiohumeral' OR 'epycondylitis humeri' OR 'external humeral epicondylitis' OR 'humeral epicondylitis' OR 'humerus epicondylitis' OR 'lateral elbow tendinopathy' OR 'lateral elbow tendinosis' OR 'lateral elbow tendonopathy' OR 'lateral epicondylitis' OR 'lateral humeral epicondylitis' OR 'radiohumeral bursitis' OR 'radiohumeral epicondylitis' OR 'tennis arm' OR 'tennis elbow' OR 'medial epicondylitis'/exp OR 'epicondylitis medialis' OR 'golfer arm' OR 'golfer elbow' OR 'medial elbow tendinopathy' OR 'medial elbow tendinosis' OR 'medial epicondylitis' OR 'medial humeral epicondylitis'.
#2 'intraarticular drug administration'/exp OR 'drug administration, intraarticular' OR 'injection, intraarticular' OR 'injections, intra-articular' OR 'intra-articular administration' OR 'intra-articular delivery' OR 'intra-articular drug administration' OR 'intra-articular drug delivery' OR 'intra-articular infusion' OR 'intra-articular injection' OR 'intra-articular injections' OR 'intra-articular medication' OR 'intra-articular treatment' OR 'intraarticular administration' OR 'intraarticular delivery' OR 'intraarticular drug administration' OR 'intraarticular infusion' OR 'intraarticular injection' OR 'intraarticular medication' OR 'intraarticular treatment' OR 'intracoxal drug administration' OR 'joint infusion' OR 'joint injection' OR 'treatment, intraarticular'.
#3 'hyaluronic acid'/exp OR 'adant' OR 'adant dispo' OR 'amo vitrax' OR 'amvisc' OR 'amvisc plus' OR 'arthrease' OR 'artz' OR 'biolon' OR 'bionect' OR 'clearvisc' OR 'duovisc' OR 'durolane' OR 'eyecon' OR 'go-on (drug)' OR 'halonix' OR 'healon' OR 'healon gv' OR 'healon yellow' OR 'healon5' OR 'healonid' OR 'hialid' OR 'hyalcon' OR 'hyalein' OR 'hyalgal' OR 'hyalgan' OR 'hyalovet' OR 'hyalubrix' OR 'hyaluronan' OR 'hyaluronate' OR 'hyaluronate sodium' OR 'hyaluronic acid' OR 'hyaluronic acid component' OR 'hyladerm' OR 'hylaform' OR 'hylan g f 20' OR 'hylan g-f 20' OR 'hylartin v' OR 'hylo-comod' OR 'hylumed' OR 'hyruan' OR 'ialugen' OR 'juvederm' OR 'lagricel ofteno' OR 'laservis' OR 'me 3710' OR 'monovisc' OR 'na hylan' OR 'na-hylan' OR 'nrd 101' OR 'nrd101' OR 'ophthalin' OR 'ophthalin plus' OR 'orthovisc' OR 'ostenil' OR 'perlane' OR 'potassium hyaluronate' OR 'provisc' OR 'radiaplexrx'. OR 'restylane' OR 'restylane lyft' OR 'si 4402' OR 'sinovial' OR 'sl 1010' OR 'sodium hyaluronate' OR 'sperm select' OR 'supartz' OR 'suplasyn' OR 'synocrom' OR 'synojoynt' OR 'synvisc' OR 'teosyal' OR 'triluron' OR 'unihylon' OR 'viscoseal' OR 'vismed' OR 'vitrax'.
#4 'corticosteroid'/exp OR 'adrenal cortex hormone' OR 'adrenal cortex hormones' OR 'adrenal cortical hormone' OR 'adrenal cortical hormones' OR 'adrenal cortical steroid' OR 'adrenal steroid' OR 'adrenal steroid hormone' OR 'adreno cortical steroid' OR 'adreno corticosteroid' OR 'adrenocortical hormone' OR 'adrenocortical steroid' OR 'adrenocorticosteroid' OR 'cortical steroid' OR 'cortico steroid' OR 'corticoid' OR 'corticosteroid' OR 'corticosteroid agent' OR 'corticosteroid calcium' OR 'corticosteroid hormone' OR 'corticosteroids' OR 'corticosteroids, inhalation' OR 'corticosteroids, ophthalmic' OR 'corticosteroids, otic' OR 'corticosteroids, systemic' OR 'corticosteroids, topical' OR 'dermocorticosteroid' OR 'fluorinated corticosteroid'.
#5 #3 OR #4.
#6 #1 AND #2 AND #5 = #6 AND [embase]/lim NOT ([embase]/lim AND [medline]/lim) = 23

**Table:**

**PORTAL REGIONAL BVS**
#1 MH:"Cotovelo de Tenista" OR (Tennis Elbow) OR (Epicondilite Lateral) OR (Epicondilite Lateral do Úmero) OR MH:C05.651.869.435.500$ OR MH:C26.088.134.750$ OR MH:C26.874.800.500.500$ OR MH:»Tendinopatia do Cotovelo» OR (Elbow Tendinopathy) OR (Cotovelo de Golfista) OR (Epicondilite Medial) OR MH:C05.651.869.435$ OR MH:C26.088.134.625$ OR MH:C26.874.800.500$
#2 MH:"Injeções Intra-Articulares" OR (Injections, Intra-Articular) OR (Injeção Intra-Articular) OR MH:E02.319.267.530.380$
#3 MH:"Ácido Hialurônico" OR (Hyaluronic Acid) OR (Hialuronan) OR MH:D09.698.373.475$
#4 MH:"Corticosteroides" OR (Adrenal Cortex Hormones) OR (Corticoesteroides) OR (corticoide) OR (corticoides) OR (Corticosteroide) OR (Hormônio Adrenocortical) OR (Hormônio Córtico-Adrenal) OR (Hormônio do Córtex Ad-Renal) OR (Hormônio do Córtex Adrenal) OR (Hormônio do Córtex das Ad-Renais) OR (Hormônio do Córtex das Glândulas Ad-Renais) OR (Hormônio do Córtex das Glândulas Suprarrenais) OR (Hormônios Adrenocorticais) OR (Hormônios Córtico-Adrenais) OR (Hormônios do Córtex Ad-Renal) OR (Hormônios do Córtex Suprarrenal) OR (Hormônios do Córtex das Ad-Renais) OR (Hormônios do Córtex das Glândulas Ad-Renais) OR (Hormônios do Córtex das Glândulas Suprarrenais) OR (Hormônios do Córtex das Suprarrenais) OR MH:D06.472.040$
#5 #3 OR #4
#6 #1 AND #2 AND #5 = 4
